# COGNITIVE AGING IN OLDER MINORITY POPULATIONS: LONGITUDINAL EVIDENCE FROM U.S. CHINESE OLDER ADULTS

**DOI:** 10.1093/geroni/igz038.3001

**Published:** 2019-11-08

**Authors:** XinQi Dong, Melissa Simon

**Affiliations:** 1 Rutgers Institute for Health, Health Care Policy and Aging Research, New Brunswick, United States; 2 Northwestern University, Chicago, Illinois, United States

## Abstract

The increasing diversity in the aging population warrants systematic investigations regarding ethnic differences related to cognitive aging and ethnicity-unique risk factors. However, due to the great paucity of population-based longitudinal data on cognitive aging in racial/ethnic minority populations, our knowledge in this area remain limited. The purpose of this symposium is, therefore, to examine various psychological, socio-cultural, and physical factors associated with cognitive aging among U.S. Chinese older adults, representing one of the biggest and fastest growing older minority populations nationally. Using longitudinal data from a population-based prospective cohort study, namely The Population Study of ChINese Elderly in Chicago (PINE) with a sample size of 3,157, this symposium presents findings from five research projects. Session 1 investigates the relationship between psychological well-being and change of cognitive function over four years. Session 2 and 3 examine the relationships between two socio-cultural factors and cognitive function. Specifically, session 2 investigates the associations between immigration-related factors and the incidence of cognitive impairment. Session 3 explores the relationship between cognitive function and Tai-Chi practice. Session 4 and 5 examine the relationships between two physical health indicators and cognitive aging. Specifically, session 4 examines the relationship between physical function and change of cognitive function over two years. Session 5 explores the association between body mass index and cognitive function decline over two years. Taken together, this symposium aims to further our knowledge of cognitive aging among ethnically/culturally diverse populations. The research findings will identify unique factors related to cognitive aging in older minority populations.

